# Advancing drug development with “*Fit-for-Purpose*” modeling informed approaches

**DOI:** 10.1007/s10928-025-09995-2

**Published:** 2025-09-15

**Authors:** Jennifer Sheng, Tongli Zhang

**Affiliations:** 1 College of Pharmacy, University of Michigan, Ann Arbor, MI 48109 USA; 2 Department of Pharmacology, Physiology, and Neurobiology, College of Medicine, University of Cincinnati, Cincinnati, OH 45219 USA

## Abstract

Model-informed Drug Development (MIDD) is an essential framework for advancing drug development and supporting regulatory decision-making. The current review presents a strategic blueprint to closely align MIDD tools with key questions of interests (QOI), content of use (COU), and model impact across stages of development —from early discovery to post-market lifecycle management. To demonstrate how the strategy works, we have also provided examples of how the MIDD tools can be applied to enhance the target identification, assist with lead compound optimization, improve preclinical prediction accuracy, facilitate First-in-Human (FIH) studies, optimize clinical trial design including dosage optimization, describe clinical population pharmacokinetics/exposure-response (PPK/ER) characteristics, and support label updates during post-approval stages. Additionally, the roles of some commonly used modeling methodologies, such as quantitative structure-activity relationship (QSAR), physiologically based pharmacokinetic (PBPK), semi-mechanistic pharmacokinetics/pharmacodynamics (PK/PD), PPK/ER, and quantitative systems pharmacology (QSP), are highlighted. What is more, we also explored the evolving role of MIDD in the context of emerging technologies, such as artificial intelligence (AI) and machine learning (ML) approaches. Further, MIDD utilities in development and regulatory evaluation of 505(b) (2) and generic drug products, as well as practical considerations of MIDD in regulatory interactions and asset acquisitions, are briefly discussed. At the end of the review, we briefly addressed the potential challenges faced by MIDD, such as lack of appropriate resources and slow organizational acceptance and alignment, as well as our perspectives on future opportunities of how MIDD could be further expanded.

## Introduction

Model-informed Drug Development (MIDD) is an essential framework in both advancing drug development and in supporting regulatory decision-making. MIDD plays a pivotal role in drug discovery and development by providing quantitative prediction and data-driven insights that accelerate hypothesis testing, assess potential drug candidates more efficiently, reducing costly late-stage failures, and accelerating market access for patients. Evidences from drug development and regulatory approval have demonstrated that a well-implemented MIDD approach can significantly shorten development cycle timelines, reduce discovery and trial costs, and/or improve quantitative risk estimates, particularly in facing the development uncertainties [1]. Furthermore, MIDD increases the success rates of new drug approvals by offering a structured, data-driven framework for evaluating safety and efficacy throughout the entire drug development process.

The history of MIDD has significantly benefited from collaborative efforts in quantitative analyses guided by both the pharmaceutical sectors, regulatory agencies, as well as academic innovations [2–6]. To standardize the MIDD practices across different countries and regions, the International Council for Harmonization (ICH) has expanded its guidance including MIDD, namely the M15 general guidance [6]. This global harmonization promises to improve consistency among global sponsors in applying MIDD in drug development and regulatory interactions, thus bears the potential of promoting more efficient MIDD processes worldwide [6–9].

We have based our perspectives on the five-stage structure defined by the Food and Drug Administration (FDA), including drug discovery, preclinical testing, clinical trials, regulatory approval, and post-market surveillance [10]. As the general structure is used to facilitate the discussions, readers should be aware that other regulators, such as EMA/PMDA/NMPA/others, may offer their own perspectives on drug development in their corresponding regulatory regions.

Meanwhile, we would also like to remind readers that a growing number of quantitative approaches are emerging, offering comprehensive methodologies for data analysis, interpretation, and clinical application within defined contexts of use (COU). These approaches support more effective decision-making throughout the drug development lifecycle, accelerate the advancement of development pipelines, and enhance the delivery of innovative therapies to patients. Accordingly, we invite the community to further expand, refine, and improve upon the pragmatic MIDD strategies outlined in this review.

In this review, we sketched out a” fit for purpose” strategic roadmap on how to select MIDD tools to meet the needs of different stages of drug discovery and development, and to answer key scientific and clinical questions, in the content of use. The fit-for-purpose (FFP) indicates that the tools need to be well-aligned with the “Question of Interest”, “Content of Use”, “Model Evaluation”, as well as “the Influence and Risk of Model” in presenting the totality of MIDD evidence and the subsequent regulatory review [11]. FDA FFP initiative offers it as a regulatory pathway, with “reusable” or “dynamic” models. Successful applications include dose-finding and patient drop-out across multiple disease areas [12]. In contrast, a model or method is not FFP when it fails to define the COU, the data quality, and the model verification, calibration, validation and model interpretation. Additionally, oversimplification, lack of data with sufficient quality or quantity, or unjustified incorporation of complexities, might also render the model not FFP. For example, a machine learning model trained on a specific clinical scenario may not be” fit for purpose” to predict a different clinical setting.

The overarching concepts and principles in this review provide a broad overview with successfully applied examples, and we hope to inspire readers with interests to tailor their own MIDD practices to boost success via the “*fit-for-purpose*” approach. Individualization will be needed to address the challenges of modern pharmaceuticals projects, such as the emergence of new modalities, changes of standard of care, and combination therapies. “Fit-for-purpose” implementation, strategically integrated with the scientific principles, the clinical evidence and the regulatory guidance, along with the quantitative methodologies, promises to empower development teams to shorten development timelines, reduce the cost, and ultimately benefit patients.

## Five stages of drug development

Drug development follows a structured process with five main stages, each playing an important role in ensuring a new drug is safe and effective [10].

1. **Discovery**: Researchers identify disease targets and test compounds to find potential drug candidates that can interact effectively with those targets.
2. **Preclinical Research**: Promising candidates are tested in laboratory and animal studies to evaluate their biological activity, potential benefits, and safety before they are tested in humans.
3. **Clinical Research**: The drug is tested in humans in three phases. Phase 1 assesses safety in a small group. Phase 2 evaluates effectiveness and side effects in patients. Phase 3 confirms benefits and compares the new drug with existing treatments in a larger population.
4. **FDA Review**: If clinical trials are successful, the drug developer submits all data to the FDA. The FDA reviews the results, manufacturing details, and proposed labeling before deciding on approval.
5. **Post-Market Monitoring**: Once approved, the drug continues to be monitored for safety in real-world use, with ongoing data collection to detect any unexpected issues.

Although this process is standardized, using MIDD effectively requires experienced teams with multidisciplinary expertise. The collective insights are essential to choose and apply the right modeling tools at the right time to support decisions and improve outcomes for patients.

The questions faced by drug development teams are often diverse, calling for flexible application of available MIDD approaches and tools. For example, some key questions could include:

- “Which models will provide the best insights for this indication at this stage?”
- “We have very limited patient data in FIH trial, how can we accelerate via MIDD?”
- “Why are certain MIDD approaches needed for drug product A but not B?”
- and “We have the models and simulation results, how to incorporate them into the overall development strategy?”

By addressing the questions of interest in the most relevant COU, the strategic and scientific leaders, collaborating closely with cross-functional teams, including but not limited to pharmacometricians, pharmacologists, statisticians, clinicians, and regulatory colleagues, together ensure that MIDD tools not only make the timeline shorter but also improve the probability of success via more quantitative assessment. Ultimately, a deeper understanding of MIDD and its “*fit-for-purpose*” applications would benefit patients with unmet medical needs with new and alternative treatment options.

## Objectives of the current review

In this review, we would like to achieve the following objectives:

1. To provide a high-level perspective on the important role of MIDD in optimizing each stage of drug development, and to outline the frequent MIDD quantitative tools (Table 1), and to elucidate their general utilities addressing diverse relevant questions of interests.
2. To present a roadmap illustrating how commonly utilized PMx tools (Fig. 1) align with development milestones, guiding the progression from early discovery through regulatory approval, and ensuring that methodologies are appropriately matched to QOI in the COU.

**Fig. 1:**
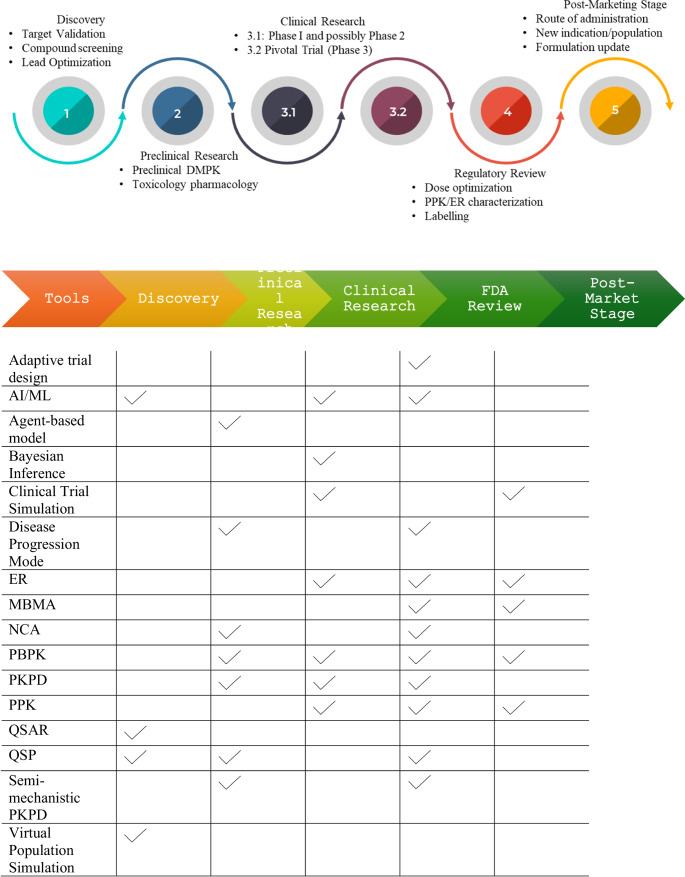
Illustration of commonly used MIDD tools across new drug discovery and development
3. To discuss the MIDD application in 505(b) (2), generic, and practice.
4. To list current challenges and opportunities.

In Table 1, we summarize some key tools that are frequently applied in MIDD.

**Table 1:** Commonly utilized MIDD tools

Tools	Description
Quantitative Structure-Activity Relationship (QSAR)	Computational modeling approach to predict the biological activity of compounds based on their chemical structure.
Physiologically Based Pharmacokinetic (PBPK)	Mechanistic modeling approach that focuses on a mechanistic understanding of the interplay between the physiology and drug product quality [13].
Non-compartmental Analysis (NCA)	A model-independent approach that estimates drug exposure and clearance parameters directly from rich plasma concentration-time data without assuming body compartments.
Semi-Mechanistic PK/PD	A hybrid modeling approach combining empirical and mechanistic elements to characterize drug pharmacokinetics (PK) and pharmacodynamics (PD).
Population Pharmacokinetics (PPK)	Well-established modeling approach to explain some of variability in drug exposure among individuals for population(s) [14].
Exposure-Response (ER)	Analysis of the relationship between a defined drug exposure and its effectiveness or adverse effects (safety) of drugs [15].
Quantitative and Systems Pharmacology/Toxicology (QSP/T)	Integrative modeling framework combining systems biology, pharmacology/toxicology, and specific drug properties, to generate mechanism-based prediction on drug behavior, treatment effects, and potential side effects.
Artificial Intelligence (AI) in MIDD	AI-driven and machine-based system to analyze large-scale biological, chemical, and/or clinical datasets for defined objective, make predictions, recommendations, or decisions influencing real or virtual environment.
Machine Learning (ML) in MIDD	ML refers to set of techniques that can be used to train AI algorithms to improve performance at a task based on data, and to enhance drug discovery [16], predict ADME properties, and optimize dosing strategies.
First-in-Human (FIH) Dose Algorithm	Model-based dose prediction strategies, including toxi-kinetic PK, allometric scaling, QSP and semi-mechanistic PK/PD, and NCA to determine the starting dose and the subsequent dose escalation in human trial.
Model-Integrated Evidence (MIE)	Use of PBPK and other computational models to generate evidence for generic drug product development in bioequivalence.
Bayesian Inference	Probabilistic modeling approach that integrates prior knowledge with observed data for improved predictions.
Adaptive Trial Design	Model-based approach to dynamically modify clinical trial parameters based on “real-time” accumulated data.
Clinical Trial Simulation	The use of mathematical and computational models to virtually predict trial outcomes, optimize study designs, and explore potential clinical scenarios before conducting actual clinical trials.
Virtual Population Simulation	A computational modeling technique that creates diverse, realistic virtual cohorts of individuals and subgroup populations, to predict and analyze biological, pharmacological, or clinical outcomes under varying conditions.
Model-Based Meta-Analysis (MBMA)	A quantitative approach that integrates data from multiple literature reported and proprietary studies using mathematical models to predict clinical outcomes.
MIDD in Generic Drug Development	Use of PBPK and other models to support bioequivalence assessments and regulatory approval of generic drugs.
Post-Market Label Update	Use of ER and PBPK models to support changes in dosing regimens and new indications for approved drugs.
Regulatory MIDD Pathway	Engagement with FDA, EMA and other health authorities through MIDD Paired Meetings and consultations on MIDD approach in drug development and regulatory review

In Fig. 1; Table 2, we illustrate and summarize the stages of drug development alongside relevant modeling tools. A common and practical question faced by development teams is: *“Which models will provide the most useful insights for this indication at this stage?”* The answer depends on selecting modeling strategies that are *fit-for-purpose*—aligned with the specific phase of development, intended indication, and the key scientific or regulatory questions being addressed.

For example, during early discovery and lead optimization, QSAR models, AI/ML tools for target prediction, or QSP frameworks may be most valuable for generating translational insights. In the preclinical stage, PBPK and semi-mechanistic PK/PD models, combined with allometric scaling, can be employed to project human pharmacokinetics and inform first-in-human (FIH) dose selection. During early clinical trials, Bayesian adaptive designs, population PK (PPK) models, and exposure–response (ER) models are particularly useful for characterizing variability and linking exposure to early safety or efficacy signals.

As development progresses, the modeling approach must evolve accordingly—from exploration to decision-making. PPK/ER models are frequently used for dose selection and justification in pivotal trials, while disease progression and model-based meta-analyses (MBMA) can support long-term efficacy projections or indirect comparisons. In the post-market phase, virtual population simulations and PBPK-based drug–drug interaction (DDI) models are often used to refine product labeling and meet post-approval commitments.

Given the complexity of drug development, along with organizational differences in strategy, resources, and timelines, we encourage developers to adopt *fit-for-purpose MIDD practices*—selecting and tailoring models that are most appropriate for their context. This approach ensures that modeling efforts are both efficient and impactful, supporting informed decisions and improving the likelihood of success across the product lifecycle.

(a) Models are listed in the alphabetical order; (b) including but limited to the models listed in Fig. 1. (c) FDA review various models across therapeutic areas, modalities and the unmet medical needs.

**Table 2:** New drug development phases and the corresponding modeling tools

Drug Development Phase	Key Objectives	Frequently Used Modeling Tools
1. Discovery	Identify drug targets and optimize lead compounds	- QSAR for structure-activity relationship
		QSP for target and lead selection
		- ML for preclinical ADME prediction
		- AI for target identification and lead asset selection
2. Preclinical Research	Evaluate safety, ADME properties, and toxicity	- NCA for characterizing preclinical PK in various toxicology species
		- PBPK modeling for predicting human PK and exploring FIH formulations
		Semi-mechanistic PK/PD for predicting minimal human efficacious dose
		QSP/T for the starting dose in FIH
		AI/ML for identifying potential toxicity outcomes
3. Clinical Research	Assess safety, efficacy, and dose-response in humans	-QSP- for MOA and dose selection
		Bayesian Inference for dose escalation and DLT
		- PPK for PK characterization in HV and in patients
		semi-mechanistic PKPD model for characterizing exposure-pd biomarkers relationship
		ER modeling for characterizing the exposure-efficacy and exposure-safety relationship
		PBPK for formulation change, update on route of administration, and potential dose adjustment in pediatric, geriatric and other special populations
		disease progression modelling for monitoring and predicting the disease
		agent-based models to connect multi scales
		AI/ML for patient selection
4. FDA Review	Ensure safety, efficacy, and optimized dosing	- Hierarchical Pharmacometric Modeling
		- Bayesian Inference for dose optimization
		PPK and ER for dose justification and recommendation
		- MIE for regulatory decisions
		- AI/ML for patient population selection
5. Post-Market Surveillance	Monitor safety, optimize dosing, expand indications	- PPK and ER modeling for dosage adjustment
		- PBPK for DDI and special population
		- Adaptive trial design for new indications Virtual population and clinical trial simulation for formulation bridging and change of route of administration
		- MBMA and AI/ML for real-world evidence analysis

### Discovery

During the discovery phase, MIDD leverages computational modeling and simulations to streamline the target identification and validation, as well as the lead compound optimization and nomination. By integrating multimodal data sources and predictive analytics, MIDD enables a more informed and strategic approach to discovering novel therapeutic candidates.

#### Target identification and validation

MIDD uses advanced technologies like artificial intelligence (AI), machine learning (ML), multi-omics data, and bioinformatics to analyze multi-scale biological systems. AI and ML algorithms, building upon traditional quantitative structure-activity relationship (QSAR) models, can predict potential drug targets by analyzing large datasets to identify patterns and correlations that conventional methods might overlook [17–19]. Omics technologies, including genomics, proteomics, and metabolomics, provide deep insights into biological pathways and disease mechanisms, enabling more precise and effective target selection through computational modeling [20, 21]. QSP, with their mechanism-based nature, can also be helpful in target validation.

#### Lead compound optimization and nomination

MIDD uses AI/ML models to predict the preclinical ADME properties of potential compounds. These models allow for rapid screening of extensive chemical libraries, significantly reducing time and resources needed for experimental testing. Enhanced QSAR models integrated with AI/ML techniques offer better predictive accuracy by considering a broader range of molecular descriptors and biological interactions. This accelerates the optimization of lead compounds, enabling early identification of candidates with optimal therapeutic profiles.

As real-world examples, the collaboration between Insitro and Bristol-Myers Squibb (BMS), showcased how ML-driven drug discovery promises to disentangle the complexity of disease (e.g., amyotrophic lateral sclerosis, ALS), and identify novel genetic targets, to potentially discover disease-modifying medicine [22]. The proprietary platform, Insitro Human (ISH), combines induced pluripotent stem cell derived disease modeling, ML analysis, human genetics and genomics to support in vitro models, hence promising to identify disease progression and patient segments, and discover potential targets [23]. Similarly, ML has been used to determine new ALS-associated genes, potential targets, critical biomarker pathways, and their associations with the disease subtypes and tissue samples [24, 25]. As another example, AstraZeneca’s collaborations with numerous external digital partners demonstrate how MIDD strategies could enhance drug discovery pipelines and highlighted the growing industry-wide adoption of these technologies [26]. Case studies of some of the most demanded drug products further illustrate the benefits of MIDD in drug discovery. For example, in the development of Semaglutide, structure modeling of the receptors provided essential insights into its ligand recognition and activation [27, 28]. Specifically, the authors reported the high-resolution crystal structure with molecular details between the ligand and the receptor, revealing major confirmational changes in secondary structure during the binding and key interactions with the peptide ligand. They further identified a dual-binding trigger model [28]. The development of EGFR (Epidermal Growth Factor Receptor) inhibitors also demonstrated the power of theoretical analysis and modeling [29, 30]. Notably, guided by the QSAR modeling along with a glutathione-based assay, a series of EGFR inhibitors were designed and synthesized to target a cystine residue in the ATP binding site, showing improved activity to overcome the mutations by gefitinib and erlotinib [30]. These model-informed insights facilitated the design and optimization of effective inhibitors, predicting their binding affinities and immunomodulatory effects, which accelerated their progression from discovery to clinical application.

### Preclinical research

The Preclinical Research phase focuses on assessing ADME properties of a drug, and preclinical toxicology assessment. PBPK modeling is an advanced tool used to simulate and predict the ADME properties of a drug in humans. By integrating detailed physiological, biochemical, and drug-specific data, PBPK models help researchers understand a drug’s distribution and elimination processes, which is invaluable for translating preclinical findings into human contexts. In addition to PBPK modeling, semi-mechanistic PK/PD modeling plays a vital role in drug development. This approach combines empirical data with mechanistic insights, providing a nuanced understanding of the relationship between drug concentration and its pharmacological effects. Semi-mechanistic PK/PD models, including both efficacy and toxicity, offer a flexible framework that adapts to varying levels of biological complexity, enhancing predictions of human PK and optimizing dose selection.

A primary application of these modeling techniques is projecting human pharmacokinetics from preclinical data. By leveraging PBPK and semi-mechanistic PK/PD models, researchers can predict how a drug is likely to behave in humans, including its absorption, distribution, metabolism, and elimination profiles. This information is essential for determining the starting dose for FIH trials [31, 32]. Accurate dose projections reduce the risk of adverse effects while ensuring therapeutic levels of the drug are achieved in the human body [33]. Ultimately, the goal of ADME assessment, PBPK modeling, and semi-mechanistic PK/PD modeling is to translate preclinical findings into a safe and efficacious dose for humans. By integrating data from various modeling approaches, researchers can identify a dose that is both safe and effective for the target population, thereby ensuring that new therapies provide real-world benefits to patients.

QSP models play a significant role in preclinical research by integrating biological, pharmacological, and mathematical principles to predict drug effects within biological systems. By leveraging QSP models in preclinical drug development, researchers can better optimize dosing regimens, discover reliable biomarkers, and predict efficacy and toxicity profiles, significantly reducing clinical trial failures and enhancing informed study designs throughout the pharmaceutical development process.

With their high potential, QSP modeling has been increasingly applied in the pharmaceutical industry [34]. For the research of CNS diseases, QSP modeling has been recognized as an powerful extension of the more traditional modeling methods [35]. Additionally, QSP models have been developed to evaluate the cardiovascular safety drugs was and enable risk mitigation [36].

### Clinical research

FIH studies represent a major milestone in drug development, marking the first time a new drug is investigated in humans. These studies assess the safety, PK, and possibly exploratory efficacy and PD of a drug, in healthy volunteers or patients. In recent years, (QSP) modeling has gained prominence in FIH studies. By offering a comprehensive understanding of the intricate interactions between in vitro cellular data, preclinical toxicity and PK, the underlying disease mechanisms, and the drug properties, QSP may support the selection of substantially higher starting doses for FIH trials compared to traditional methods like minimum anticipated biological effect level (MABEL). The predicted starting doses were 5 mg/kg and 0.045 to 0.1 mg/kg, using QSP and MABLE methods, respectively, with 50–100 folds higher [37].

This approach was accepted by FDA and Australian Human Research Ethics Committee, and accelerated the dose escalation of FIH trial, reducing the sub-efficacious doses to patients and saving valuable time and cost. Additionally, modified MABEL approach was proposed, using the most relevant rather than the most sensitive measure of pharmacological activity [38, 39]. Specifically, the median EC_50_ from the primary cell killing, rather than using EC10-30% in the most sensitive cell assay, improving the starting dose to be closer to the efficacious dose to patients [38]. By integrating biological, disease-specific data, and drug pharmacological data, QSP models also promise to enhance the ability to predict drug responses, optimize dosing strategies, and reduce the risk of adverse events during early trials.

In early-phase clinical development, a common challenge arises: “We have very limited patient data in FIH trial—how can we accelerate via MIDD?” Quite often, it would benefit from maximizing prior knowledge. Baysesian model-based adaptive design is increasingly applied, aiming to reduce the sample size in human trials, by leveraging data derived from in vitro, preclinical or human data. integrating in vitro pharmacology, preclinical PKPD, and early clinical human data (ref: current reference list 39). Additionally, mechanistic models such as PBPK and QSP, along with virtual population simulation, would derisk the uncertainties [40–42]. With increased number of participants, PPK plays a more vital role in FIH studies and beyond, describing the typical pk parameters and quantifying the variability in drug concentrations across individuals. PPK modeling helps to identify covariates such as age, weight, renal function, or genetic factors that affect the drug exposure measures. More importantly, drug exposures with high variability and/or non-linear pk would necessitate the exposure-safety analysis than the conventional dose-limiting toxicity approach. Quantifying the variability or uncertainties further allows for optimizing dose and dosing regimens with FIH trials, where the enrolled patients are “all comers”, in oncology therapeutic area, thereby enhancing drug benefit risk ratios. For example, exposure-toxicity guided Bayesian design vs. the dose-toxicity design was postulated in a Phase I dose escalation trial, with 15 patients administered with four different dose levels. The work demonstrated that exposure-based models would improve the selection of optimal dose, when the drug exhibits non-linear pk or large inter-subject variability [43]. Additionally, PPK modeling helped to determine appropriate dosing in different age groups and special populations, ensuring broad and safe vaccine deployment [44].

Exposure-Response (ER) analysis is another indispensable aspect in drug development. It involves characterizing the relationship between various drug exposures (and their efficacy or safety endpoints. ER models are essential in clinical studies to recommend RP2D dose, prior to the pivotal trial. For instance, Stelara (ustekinumab), used to treat autoimmune diseases like psoriasis and Crohn’s disease, relies on ER modeling to balance efficacy against potential side effects. For Stelara, ER analysis determined the dose that maximizes therapeutic benefits while minimizing risks [45, 46]. After the pivotal clinical trial, the PPK and ER models have been frequently applied for dose confirmation and benefit/risk assessment, along with clinical efficacy and safety data.

The integration of QSP, PPK, and ER models throughout drug development creates a robust framework for making well-informed decisions. These models allow decision makers to simulate various scenarios, predict outcomes in diverse patient populations, and support regulatory submissions. As discussed, the combination of PPK and ER models during the development of Comirnaty facilitated the rapid optimization of dosing strategies, which was critical under the accelerated timelines of the COVID-19 pandemic. Similarly, QSP models for Stelara provided a deeper understanding of its mechanism of action, enabling more refined clinical use across different inflammatory conditions. Major health authorities encourage the sponsors to discuss these MIDD approaches with the agencies in early clinical development. They have issued many draft or final guidance, published papers, hosted MIDD-centric workshops, designated regulatory pilot programs, or formed modeling & simulation working group [47–50].

In summary, FIH and the pivotal clinical trials, together with QSP, PPK, and ER models, are revolutionizing drug development by offering a precise and predictive understanding of drug behavior in humans. These models not only help the drug developers to maximize the benefit risk ratios of new therapies but also advance personalized medicine approaches. Success stories like Comirnaty and Stelara underscore the essential role these models play in bringing life-saving therapies to market and enhancing patient outcomes worldwide.

### FDA review

Applications of MIDD principles in FDA reviews started in early 1990 s and have continuously evolved to the transformative presence today [5]. The scope of MIDD applications is across all therapeutic areas, across adults and pediatric populations, and across stages of drug development such as EOPI/II meetings and the label update. With the emergence of advanced science and technologies, the methodologies have expanded from the conventional PBPK-based to further mechanistic QSP modeling, from PPK/ER based models to early adoption of AI/ML models, and from earlier applications to the standardized plan, data, analysis and reporting to regulatory bodies. Dosage selection, optimization, recommendation and confirmation, has been implemented in non-oncology disease therapeutic areas, via the totality of evidence of relevant preclinical data, doing ranging studies of clinical efficacy and safety, pharmacometrics analyses, and pivotal clinical trials. In recent years, dosage optimization becomes a mandate in oncology drug development and regulatory approval [51]. It aims to maximize the ratio between effective and safe dosage for patients, ensuring that the drug achieves its intended therapeutic effects while minimizing adverse events. The historic maximum tolerant dose (MTD) approach is more suitable for cytotoxic agents than the target or immunological therapies. Additionally, low-grade yet long-term toxicities may compromise the patient’s life of quality and reduce patient tolerability. Based on a recent IQ survey, “*getting the dosage right*” is a case-by-case approach, advocating the importance of multi-function collaborations with the focus on patient-centered paradigm [52]. The field has evolved significantly with strategic framework, deeper understanding of cancer biology, emerging treatment modalities, inherently diverse nature of tumor sub-types, therefore the non-oncology dose optimization paradigm may necessitate modifications for mostly life-threatening oncology therapeutic areas. The totality of dose optimization largely is composed of the utilization of clinical dose/exposure with the efficacy and safety is essential and quite often available for submission, while PD data at site of action is rarely available for dose selection or optimization [52]. Translational biomarkers, such as longitudinal tumor growth dynamics across clinical visits, and IL-6 release as a CRS surrogate for T-cell engagers, have provided supporting evidence for dose optimization [53, 54].

The recent methodologies include the integration of advanced tools like ML is increasingly valuable in dose optimization. ML models can analyze vast amounts of data from clinical trials, real-world evidence, and other sources to identify patterns that may not be apparent through traditional analysis. For example, during the COVID-19 pandemic, ML models helped optimize dosing regimens for various treatments, ensuring that patients received the most effective and safe doses for antiviral drugs and monoclonal antibodies [55, 56]. Additionally, AI/ML approaches, including both elastic net regression and artificial neural network, were independently applied to predict whether a patient is to benefit from Anakinra treatment, using the score rule of soluble urokinase plasminogen activator (suPAR) value of 6 ng/mL based on the SAVEMORE trial data. With 30 variables available from the baseline characteristics of the trial patient’s data, elastic new regression was to select the contributing features, and the neural network model was independently to rank the features and the cut-off values. Both methods resulted in consistent eight criteria for patient suPAR prediction. Further, the model was externally validated using SAVE trial [55, 56]. The application of ML, an emerging MIDD tool, offers a powerful predictivity of patient selection with benefits of the treatment. This AI/ML approach is especially valuable in therapeutic areas where patient responses can vary widely, especially during a global health crisis [57].

The future of MIDD, including dose optimization, lies in further integrating and refining the existing and emerging tools. As more clinical data becomes available, the role of ML and AI in dose optimization will expand. It is just the beginning, demonstrating how advanced modeling techniques and innovative technologies can lead to safer, more effective therapies for patients.

#### Post-market stage

MIDD continues even after the market authorization, and the earlier applications are the post-marketing commitment/requirement implemented for dose optimization for several approved drug products, such as ponatinib [58] and ceritinib [59].

Different types of modeling play diverse role in post-market stage by enabling the continuous evaluation of a drug’s safety, efficacy, and real-world effectiveness after the initial regulatory approval. Pharmacometric models, such as PPK PKPD, and ER models, help refine dose adjustments for specific patient subgroups based on real-world variability. ML and AI-driven models analyze vast post-marketing surveillance data, including electronic health records (EHRs) and spontaneous adverse event reports, to detect rare but serious safety signals more efficiently than traditional methods. Real-world evidence (RWE) models, incorporating data from observational studies and registries, assessing long-term treatment outcomes, adherence patterns, and comparative effectiveness in diverse populations. Systems pharmacology and mechanistic models contribute by predicting potential off-target effects or long-term biological consequences based on known drug interactions. By integrating these diverse modeling approaches, post-market studies can enhance drug safety monitoring, optimize therapeutic use, and support regulatory submission in response to emerging risks or new clinical evidence.

Recent cases include but not limited to formulation change, dosage adjustment and label update. Published examples are nivolumab (often referred to as “Nivo”) and Pembrolizumab (“Pembro”) are both immune checkpoint inhibitors used primarily to treat cancers such as melanoma, non-small cell lung cancer (NSCLC), and renal cell carcinoma. These drugs work by targeting and blocking the programmed death-1 (PD-1) receptor on T-cells. Normally, the PD-1 receptor interacts with its ligand, PD-L1, often expressed by cancer cells, leading to the suppression of the immune response. By blocking this interaction, these drugs release the “brakes” on the immune system, allowing it to recognize and attack cancer cells more effectively. This ability to enhance the immune response against cancer has made them critical components of modern oncology treatments. One example is that pembrolizumab has been particularly notable for introducing a dosing schedule of every six weeks (Q6W) instead of the more frequent every three-week interval [60, 61]. This extended dosing schedule offers several advantages, including increased convenience for patients, reduced healthcare burdens, and potentially improved patient adherence to treatment. This regime is particularly advantageous for patients requiring long-term treatment, reducing hospital visits while maintaining efficacy and safety.

In some cases, Nivolumab or Pembrolizumab may be used in combination with other types of treatments in clinical trials. Combining these immune checkpoint inhibitors can enhance anti-tumor responses, but it may also increase the risk of immune-related adverse events. The decision to use these drugs in combination depends on the specific type of cancer, the patient’s overall health, and underlying conditions that could increase side effects. Clinical trials continue to explore these combinations’ safety and efficacy for different cancers [62, 63].

Prevnar, a pneumococcal conjugate vaccine, provides protection against Streptococcus pneumoniae, the bacterium responsible for infections like pneumonia, meningitis, and sepsis [64, 65]. When patients are receiving immunotherapy such as Nivolumab or Pembrolizumab, vaccination strategies must be carefully considered due to the immune system modulation these treatments cause. Oncologists often work closely with healthcare providers to ensure cancer patients are appropriately vaccinated, considering the timing and type of vaccines to optimize cancer treatment while maintaining overall health [64, 65].

## MIDD in 505(b)(2) and generic drug development

MIDD principles are not only applied in 505(b) (1), but also increasingly applied in 505(b) (2) and 505(j) applications. A 505(b) (2) exhibits a hybrid state between new and genetic drug product applications.

MIDD has gained significant tractions in 505(b) (2) applications to guide formulation optimization and the selection of to-be-marketed formulation, by incorporating “fit-for-purpose” modeling and simulations, into the integration of the existing literature and the original sponsor data. Commonly, the practice would integrate data from various sources, including in vitro dissolution studies, external and internal clinical trials, then predicting the PK exposures, and/or predicting the safety and efficacy of changes, with PBPK, QSP and/or ER models. The comprehensive data and model interpretation package supports the totality evidence for the regulatory assessment. This approach is especially beneficial for drugs undergoing formulation changes or targeting special populations, where clinical trials could be potentially waived or sample-size reduced, based on the existing in vitro and/or clinical data, robust pharmacometrics analyses and scientific rationale. Approval of canagliflozin and metformin hydrochloride FDC is such an example. The ER analyses were used to bridge the efficacy of canagiflozin between the once-daily and twice-daily dosing regimen. Additionally, the time profiles between the canagliflozin exposure and HbA1C were linked using an ER model, considered of the baseline patient characteristics. The subsequent clinical trial simulation supported the approval of the FDC formulation [66].

Quantitative modeling has played an integrable role in 505(j) or ANDA/biosimilar product development and global regulatory submissions, ranging from the bioequivalence study design, demonstration of BE, supporting the regulatory assessment or approvals across narrow therapeutic index and highly variable drug products [67, 68]. MIDD framework further enhances the utilities of modeling and simulations in the modern paradigm of generic drug development, alleviating the burden of in vivo BE studies, accelerating the market access, and offering patients clinically equivalent yet affordable alternatives. Among many choices of models, PBPK models are often applied to simulate a drug’s absorption, distribution, metabolism, and excretion, along with pharmacodynamic biomarkers, offering insights of the pk and/or PD endpoints between the test and reference drug products, without or with limited in vivo studies. This approach has been applied to both systematically and locally acting products. Examples are ocular acting drug e.g. Besivance^®^ 0.6% [69], topical product e.g., the topically applied cream such as Voltaren^®^ topical gel 1% and orally inhaled such as bronchodilators [70]. Additionally, PBPK models are particularly valuable in assessing the effects of food on drug absorption, simulating the bioequivalence under fasting and fed conditions. Additionally, MIE (model integrated evidence) referring to use of model generated information provides confirmatory evidence to BE endpoints assessment [71]. The pilot MIE program offers meeting opportunities between the generic applicants and FDA generic office to foster the early interactions, and to focus on employing MIE approaches in establishing BE with scientific-driven topics, across multiple drug products. In theory, reusing the original model developed by the initial sponsor, to the multiple generic applications by the new sponsors, with updated datasets, might be possible, however, practicality remains almost impossible, for example, the patient or even visit-level data sharing may face comprehensive considerations.

These MIDD approaches provide a faster, more cost-effective pathway to evaluate or demonstrate whether the generic test products perform similarly to the reference list drug product (RLD). By incorporating these models, many time-consuming and expensive clinical studies can be replaced by robust models and simulations, allowing generics to reach the market faster than in vivo BE clinical trials.

## Practical considerations in regulatory interactions

The practical application of MIDD spans various stages of drug development, from study design to product approval, as well as life-cycle management updates, such as dose adjustments or alteration of the route of administration [3, 4, 48, 55, 72]. FDA recognizes the significance of MIDD and designates it as a regulatory pathway to allow multiple engagements between the sponsor and the agency i.e. the FDA’s Model-Informed Drug Development Paired Meeting Program. This pathway allows sponsors to interact with the FDA throughout the drug development process, discussing and refining development strategies, and aligning development strategies with regulatory expectations. MIDD provides a platform to address key concerns early, potentially reducing development timelines and increasing the probability of regulatory successes.

Key steps in interacting with regulatory bodies, particularly the FDA, include the following:

1. Early-stage study design and data considerations for models.
2. Mid-stage evaluations of the benefit-risk profile using modeling tools.
3. Late-stage interactions such as posology updates and route of administration changes guided by MIDD principles.

For regulatory interactions, preparing for the FDA’s MIDD Paired Meeting Program involves the following:

- Presenting the scientific question of interest.
- Describing the regulatory impact.
- Outlining the type of models used, the context of their use, and the credibility assessment required to ensure the model meets regulatory standards.

For the EMA, two primary approaches facilitate interactions:

1. The EMA Innovation Task Force, which provides an early dialogue forum.
2. Scientific Advice Meetings, commonly used for discussions on study design, data use, and analysis methods, supported by working groups specializing in pharmacometrics and modeling.

## Role of MIDD in asset acquisition

In the context of asset evaluation and acquisition, MIDD is less utilized, through it would enable a quantitative framework that benefits both early and late-stage assets. MIDD offers essential tools that aid in the assessment of various aspects of drug development. Specifically for early-stage assets, such as those undergoing preclinical stage and FIH studies, MIDD tools like PBPK models with limited data, and with PPK/ER analyses with reasonable amounts of human data, are essential. These tools help not only to characterize the preclinical and clinical pharmacological properties of the drug, but also to quantitively link the MOAs, the existing observed data and the estimation of uncertainties, thus facilitating informed decisions about the intended efficacy and safety profiles in the intended indications. This incorporation of MIDD ensures more quantitative analyses in acquisition, beyond qualitative assessment.

For instance, the acquisition of MORF-057 by Eli Lilly, which is under investigation in three Phase 2 studies for ulcerative colitis and one for Crohn’s disease, underscores the value of MIDD. As one of these 3 studies is using one dose level only, two other studies aim to evaluate the safety and efficacy of two or three different active dose regimens of MORF-057 in adult patients following randomized, double-blind, and placebo-controlled protocols. For such purposes, MIDD enables the precise selection and optimization of dosage regimens, ensuring the drug’s performance is in line with quantitative medicine principles. This approach not only maximizes the likelihood of clinical success but also aligns with the regulatory frameworks set forth by the FDA, helping the sponsor meet stringent FDA requirements [73–75]. Similarly, in the Genmab acquisition of ProfoundBio, the application of MIDD played a key role in optimizing the dose and schedule for the PRO1181-001 phase I/II study, which included dose escalation and expansion cohorts [76]. In this scenario, MIDD contributed significantly to estimating the potential benefit-risk ratios and determining the appropriate dosing regimen for the forthcoming pivotal trial. The license agreement option between AbbVie and Simcere Zaiming for the development of SIM0500 is another example [77]. SIM0500 is currently in Phase I trial, with Part I as dose escalation and Part II as dose optimization, in patients with relapsed or refractory multiple myeloma, in both China and US [78]. The study design incorporated the model-based approaches. Specifically, the dose escalation phase began with the accelerated phase with 1–3 patients in each dose cohort, followed by standard dose escalation of 3–6 participants, where 2-paramter Bayesian logistic regression model was applied in this part. Part II dose optimization will explore two potential dose levels, randomized at the 1:1 ratio with approximately a total of 40 participants. The adaptive Bayesian design allows for integration of safety data to estimate probability of dose-limiting toxicities, and the dose optimization allows exploratory ER analyses for special interests of efficacy and safety endpoints. Additionally, patient enrollment and data collection across counties enables ethnic sensitivity analysis, potentially supporting the development strategy as well as regulatory pathways in global setting. Therefore, it improves the asset’s valuation in the co-development process.

The MIDD framework would support the resources estimation of development cost and timeline, in the context of sponsors strategic directions. Very importantly, the MIDD is a necessity in the regulatory interactions, and what has been conducted, what data are yet to be collected, and how to meet the MIDD requirement, shall be an indispensable part of asset acquisition [79]. Hence, MIDD not only serves as a key enabler for internal assessment of early-stage drug development through robust evaluation techniques but also supports late-stage acquisition efforts beyond optimizing dose and schedule. The FDA’s endorsement or guidance of MIDD further underscores its importance for regulatory successes.

## Current challenges and future opportunities

Many excellent papers have discussed the potential of MIDD [80–82] and significantly promotes its recognition by decision makers. Despite its growing adoption, MIDD still needs to overcome several challenges to achieve its full potential, and some are sampled in Table 3. In our perspective, these challenges would be best addressed by a ongoing discussion between academic, industry and regulatory experts on MIDD.

One fundamental issue is optimizing the *fit-for-purpose* MIDD blueprint within the constraints of cost and timeline, focusing on practicality rather than perfection. Ensuring that quantitative MIDD tools are tightly aligned with specific development needs across different stages is crucial for meaningful impact. However, translating preclinical and in vitro findings into human biology remains difficult due to interspecies differences and pathophysiology. Another major challenge is handling human variability and uncertainty with limited patient data, particularly in FIH trials where patient heterogeneity is high, and data is limited especially in oncology and hematology. Additionally, some of the MIDD tools are resources demanding. Mid-sized and startup biotech companies often struggle with MIDD implementation due to a lack of strategic mapping, shortage of domain expertise, and/or insufficient computational skills necessary for executing needed modeling techniques.

Scaling MIDD approaches end-to-end and consistently across programs, while ensuring effective data-sharing platforms remains a barrier, especially in multi-stakeholder environments. One frequent operational challenge is the inconsistent application of MIDD methodologies across different assets. Teams often raise the question: **“**Why are certain MIDD approaches needed for product A but not B?” The root cause can often be traced to the absence of a systematic framework for mapping the appropriate models or tools to a product’s mechanism of action, modality, stage of development, specific internal and external decision-making needs, availability of data, and/or constrained timeline. In some cases, MIDD is underutilized due to oversimplified assumptions that fail to capture the complexity of the drug. In other cases, overly complex modeling efforts may consume resources less impactful to the advancement of program.

This underscores the necessity for clear, fit-for-purpose criteria that guide both strategic decision-making, in addition to technical execution. Developing a portfolio-wide governance approach—aligned with portfolio priority, development risk, and data access—can help ensure MIDD applications are context-appropriate, resource-efficient, and impact-aligned [1].

Organization level tools such as structured decision matrices, or cross-functional MIDD governance body, may provide consistency, transparency and rule-based accountability in how MIDD strategies are selected and deployed. Ultimately, effective MIDD use requires not just technical know-how, but cross-functional understanding and consistent decision frameworks across therapeutic areas.

Also, fostering collaborative cultures, building trust in modeling, and ensuring clear communication across scientific, clinical, and regulatory teams are essential for broader adoption. The integration of AI/ML in MIDD further necessitates harmonization and standardization across regions and regulatory frameworks. Finally, achieving competitive advantage requires organizations to strategically invest in emerging technologies while balancing regulatory acceptance and resource allocation to gain industry-wide confidence in MIDD approaches.

**Table 3:** Some current challenges faced by MIDD

Challenges	Elaboration
***Overall Challenges***	
Optimization	Effectively optimizing the “fit-for-purpose” MIDD blueprint, rather than perfection, within the constraints of cost and timeline.
Alignment with Development Needs	Tightly mapping PMx tools with specific needs across each development stage.
Scale up	Ensuring consistent effectiveness across development programs
Translational Challenges:	Translating in vitro and preclinical findings into human (or patient) biology and pathophysiology.
Handling Variabilities	Capturing variabilities and quantifying uncertainties with limited patient data.
Data Heterogeneity	Addressing the high heterogeneity of “all comers” patient data collected in FIH trials, particularly in oncology and hematology.
***Implementation in Clinical Practice faced by mid-sized and startup biotech companies***	
Lack of Strategic Mapping	Lack or short of the strategic mapping capabilities needed to guide drug development effectively.
Limited Expertise	Lack the highly specialized domain expertise and computational skills to effectively implement modeling techniques.
***Scaling up MIDD approaches end-to-end.***	
Psychological Factors	Fostering collaborative cultures, building trust with modeling, and facilitating clear communications across multi-domain stakeholders.
Collaborations	Strategic and integrated planning rather than reliance on localized expertise to ensure continuity.
Data Platform	Efficient data sharing across organizational functions in commonly accessible platforms.
***Applications of AI/ML in MIDD***	
Harmonization	Needs for standardization across regions and countries.
Competitive Advantage	Gaining the boarder acceptance and allocating resources for new technologies for competitive advantage.

Meanwhile, as MIDD continues to evolve, several opportunities arise:

1. Scale up of consistent MIDD deployment across pharma and biotech sectors.
2. Global applications of MIDD, with the forthcoming final establishment of ICH guideline on MIDD [6, 7].
3. Effective incorporation of modeling & simulation results in the overall program development strategy.
4. Incorporation of AI/ML into MIDD, enhancing predictive accuracy and providing a robust framework for drug development [83–85].

A major opportunity in advancing MIDD lies in transforming modeling outputs into actionable strategic tools. Development teams often raise the practical question: “We have the models and simulation results—how do we incorporate them into the overall development strategy?” Despite successful generation of exposure–response models, PBPK simulations, or QSP predictions, many organizations struggle with translating these results into dose selection, safety risk management, clinical trial optimization, or go/no-go decisions. To bridge this gap, the development teams may raise the QOI, which can sit in drug target or candidate election, target product profile, patient population strategies, integrated development plan, trial design scenarios, or regulatory briefing documents [86, 87]. The strategic incorporation may be further operationally enhanced by aligning the key decision milestones and the modeling timelines, via multi-functional team discussions. Organizational operation may implement MIDD checkpoints and/or cross-functional review to enable the model outputs are visible to the relevant functions. As MIDD continues to gain traction with regulators and industry, integrating into development strategy drives more quantitative-driven decisions and improves program success.

The integration of ML and AI methods potentially offers several advantages in addition to traditional approaches. For example, ML and AI can analyze vast and multimodal datasets more efficiently, identifying intricate patterns and relationships that may not be easily captured by conventional models. Unlike traditional pharmacometrics, which often relies on predefined mechanistic equations, ML can adaptively learn from data, improving predictive accuracy in cases where underlying biological mechanisms are not fully understood. These methods also enable rapid hypothesis generation, model refinement, and real-time decision support, making them valuable in optimizing dose selection, identifying biomarkers, and predicting patient-specific responses. Furthermore, AI-driven approaches might further enhance efficiency by automating labor-intensive tasks.

However, the use of ML and AI in MIDD comes with inherent risks if not applied properly. One major concern is the lack of interpretability and transparency in some ML models, particularly deep learning approaches, which can function as “black boxes” without clear mechanistic insights. This can limit regulatory acceptance and trust in AI-generated predictions. Additionally, ML models are highly dependent on the quality and representativeness of training data; biases or incomplete datasets can lead to misleading conclusions and unreliable extrapolations. Overfitting is another risk, where models may perform exceptionally well on training data but fail to generalize to new, unseen cases. If not properly validated and integrated with domain knowledge, ML-based models may produce spurious correlations rather than causal insights, leading to flawed extrapolation in drug development. Thus, while ML and AI offer powerful tools for MIDD, the future drug development might benefit the most from the integration between mechanism-based modeling and AI/ML approaches [88, 89].

In conclusion, MIDD is a powerful paradigm with promises to revolutionize drug discovery and development pipeline. The “fit-for-purpose “applications of MIDD require clear strategic vision, scientific insights, and effective execution. As MIDD tools become increasingly adopted and tightly integrated with the unique necessities across programs stages, they will continuously drive the efficiency and innovation in drug development, ultimately benefiting the patients with unmet medical needs.

## Data Availability

No datasets were generated or analysed during the current study.
